# Force-Dependent Presence of Senescent Cells Expressing Vascular Endothelial Growth Factor During Orthodontic Tooth Movement

**DOI:** 10.3390/biology15020187

**Published:** 2026-01-19

**Authors:** Yohei Morihana, Masato Nakagawa, Yue Zhou, Hidetoshi Morikuni, Zi Deng, Yoshitomo Honda, Aki Nishiura

**Affiliations:** 1 Department of Orthodontics, Osaka Dental University, 8-1 Kuzuhahanazono-cho, Hirakata 573-1121, Osaka, Japan; morihana-y@cc.osaka-dent.ac.jp (Y.M.); morikuni@cc.osaka-dent.ac.jp (H.M.); 2 Department of Oral Anatomy, Osaka Dental University, 8-1 Kuzuhahanazono-cho, Hirakata 573-1121, Osaka, Japan; dengzi1014@gmail.com (Z.D.); honda-y@cc.osaka-dent.ac.jp (Y.H.); 3 Stomatological Center, Affiliated Hospital of Yunnan University, No. 176, Qingnian Road, Kunming 650021, China; oduzhou@outlook.com

**Keywords:** mechanical force, cellular senescence, senescence-associated secretory phenotype (SASP), vascular endothelial growth factor (VEGF), endothelial cells, angiogenesis, orthodontic force

## Abstract

Orthodontic force magnitude influences angiogenesis during orthodontic tooth movement (OTM); however, the role of senescent cells remains largely unclear. This study investigated the localization of senescent cells and their expression of vascular endothelial growth factor (VEGF) during angiogenesis using a rat horizontal OTM model with different force magnitudes. Nickel–titanium coil springs exerting 60 g or 180 g of orthodontic force were applied to the maxillary first molar of 15-week-old male Sprague–Dawley rats; untreated rats served as controls. Tooth movement was evaluated by stereomicroscopy and micro-computed tomography. Senescent cells (p21, p16) and angiogenesis (CD31 and VEGF) were evaluated by multiplex immunofluorescence. Tooth movement was observed under both the 60 g and 180 g conditions. The 60 g group showed increased cellularity, vascular density, and VEGF expression, suggesting an optimal mechanical force. In contrast, the 180 g group reduced cellularity and angiogenesis, consistent with excessive force. Senescent cells were more abundant in the 60 g group, with over 40% expressing VEGF. These findings suggest that force magnitude influences the presence of VEGF^+^ senescent cells, which may be associated with the angiogenic process in OTM. This work provides insights into the mechanisms underlying optimal force in orthodontic treatment.

## Simple Summary

Mechanical force is an inducer of cellular senescence; however, the influence of orthodontic force magnitude on the presence of senescent cells in angiogenesis during orthodontic tooth movement remains unclear. In this study, we examined the presence of senescent cells and their expression of vascular endothelial growth factor (VEGF) in a rat tooth movement model with different force magnitudes. A moderate force increased both senescent cell abundance and angiogenesis, whereas a stronger force reduced these responses. Senescent cells expressing vascular endothelial growth factor appeared mainly under moderate force. These findings suggest that force magnitude influences the presence of VEGF^+^ senescent cells during orthodontic tooth movement.

## 1. Introduction

Orthodontic tooth movement (OTM) induces angiogenesis within the periodontal ligament (PDL), facilitating tissue remodeling by delivering gases and nutrients [1]. Conversely, the application of orthodontic force compresses the vasculature on the pressure side (PS), resulting in reduced perfusion and subsequent cellular injury, including apoptosis [2]. The magnitude of the orthodontic force is a crucial determinant of tissue remodeling. Optimal force promotes remodeling, whereas excessive force delays tissue remodeling [3,4,5]. Therefore, force magnitude plays a pivotal role in regulating PDL reconstruction and angiogenesis.

Angiogenesis is regulated by various signaling molecules, including vascular endothelial growth factor (VEGF), fibroblast growth factor (FGF), and transforming growth factor-β (TGF-β). Among these, VEGF acts as a central mitogen [6,7]. Upon ligand binding to receptors, VEGF initiates intracellular signaling cascades that stimulate endothelial proliferation, migration, and tube formation [8,9,10]. Vascular endothelial cells (ECs) interact closely with these signaling molecules through two primary mechanisms: paracrine activation by adjacent cells and autocrine secretion of factors such as VEGF and basic FGF, which sustain their proliferative and migratory capacities [11,12]. During OTM, VEGF upregulation promotes angiogenesis and dynamic changes in the PDL [13,14,15,16].

Mechanical stress potently triggers cellular senescence [17,18], which is characterized by irreversible cell cycle arrest. Orthodontic mechanical force has been implicated in the induction of senescent cells in vertical intrusion models [19]. Mechanical overloading has been shown to induce senescence in joints across in vivo, in silico, and human studies [20,21]. Moreover, the combination of microbial infection and mechanical stress has been shown to enhance senescence induction [22]. Collectively, these findings suggest that mechanical stress induces cellular senescence under various conditions.

Through the senescence-associated secretory phenotype (SASP), senescent cells secrete cytokines, matrix metalloproteinases (MMPs), and growth factors that influence the surrounding microenvironment [23,24]. SASP promotes remodeling, including wound healing and angiogenesis [25,26,27,28], whereas it also drives tissue destruction during chronic inflammation [29,30,31,32,33]. This dual role of SASP—beneficial in tissue remodeling, whereas harmful in chronic diseases—has recently attracted considerable attention.

Despite these insights, whether senescent cells expressing pro-angiogenic SASP factors such as VEGF emerge in a force-dependent manner during horizontal OTM and are associated with differences in angiogenic responses remains unclear. Therefore, this study aimed to clarify the localization and characteristics of senescent cells and to investigate their association with angiogenesis in a rat horizontal OTM model with different force magnitudes.

## 2. Materials and Methods

### 2.1. Animals

Twelve male 15-week-old Sprague–Dawley rats were obtained from Shimizu Laboratory Supplies (Kyoto, Japan) and used in this study. During the experimental period, the rats were maintained under controlled environmental conditions and provided with a soft diet prepared from powdered MF feed (Oriental Yeast, Tokyo, Japan) mixed with water. Rats were carefully monitored throughout the study period, and no animals met the criteria for humane endpoints. All animal experiments were performed in accordance with the guidelines approved by the Osaka Dental University Institutional Animal Care and Use Committee (approval no. 24-01015, 25-02013).

### 2.2. Establishment of the OTM Model

**Figure 1:**
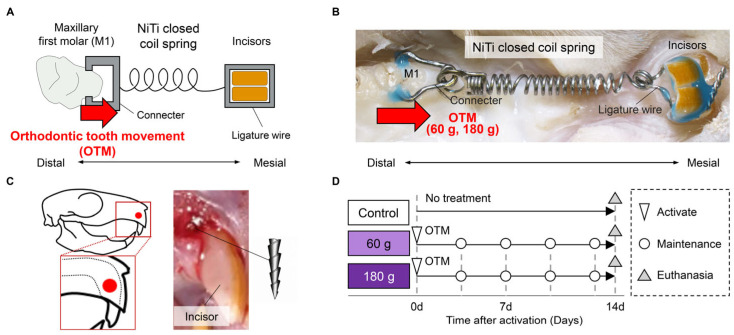
Procedure for establishing and analyzing the orthodontic tooth movement (OTM) model. (**A**) Schematic illustration of the OTM model using a nickel–titanium (NiTi) closed coil spring. M1: maxillary first molar. (**B**) Photographic image of the OTM model using a NiTi closed coil spring. (**C**) Schematic illustration and photographic image of the eruption-blocking procedure for rat incisors. (**D**) Experimental schedule for the control, 60 g, and 180 g force groups.

A lateral OTM model was established using a nickel–titanium (NiTi) closed-coil spring (509-29, Tomy International, Tokyo, Japan) connecting the maxillary first molar (M1; target tooth) and the incisors (anchorage tooth), as shown in Figure 1A,B. Before appliance placement, incisor eruption was arrested. Under general anesthesia, a 0.6 mm-diameter transosseous channel was created approximately 2 mm apical to the alveolar crest using a diamond bur, and the anchorage was secured by inserting the tip of a sectioned #35 H-file (Figure 1C). The coil spring, calibrated for its applied force magnitude using an electronic spring balance (NaRiKa, Tokyo, Japan), was attached to the M1 side with a 0.016-inch stainless steel connector and to the incisor side with a 0.010 × 0.025-inch ligature wire. Both connections were reinforced with light-cured resin cement (Solventum, Maplewood, MN, USA). Intraoperative photographs were captured with a digital camera (Tough TG-6, Olympus, Tokyo, Japan).

Twelve rats were randomly assigned to three groups: (i) control (no treatment) group, (ii) 60 g group, and (iii) 180 g group (Figure 1D), with the experimental unit defined as a single animal. After 14 days of force application, the animals were euthanized and perfused with 4% paraformaldehyde (PFA) in phosphate-buffered solution (FUJIFILM Wako, Osaka, Japan). The maxillae were harvested en bloc for subsequent analyses. No animals were excluded from the analysis, as no specific inclusion or exclusion criteria were defined. The sample size (n = 4 per group) was determined as the minimum number required for statistical analysis, based on previous studies and the 3Rs principle (Reduction).

### 2.3. Morphological Analysis

Occlusal view images were obtained using a stereomicroscope (SZ61, Olympus, Tokyo, Japan) with a digital camera (DS-Fi2/DS-L3, Nikon, Tokyo, Japan). On the occlusal view, the OTM distance was defined as the mean distance between the distal margin of M1 and the mesial margin of the maxillary second molar (M2) at three points. Micro-computed tomography (µCT) was performed using a SkyScan (Bruker, Billerica, MA, USA) system at 85 kV and 65 µA with a 0.4° rotation step. Reconstructed images were analyzed using CTvox and CTAn software (Bruker). On the axial µCT plane, the OTM distance was measured between the contact points of M1 and M2, identified at the maximum crown contour in the sagittal view.

### 2.4. Histological Analysis

The maxillary specimens were fixed with 4% PFA for 24 h at 4 °C, decalcified in 0.5 M ethylenediaminetetraacetic acid (EDTA; FUJIFILM Wako) for 2 weeks at 4 °C, and cryoprotected in 10–30% sucrose. Then, samples were embedded using Super Cryoembedding Medium (SECTION-LAB, Yokohama, Japan). Frozen blocks were sectioned at 7 µm with a cryostat (CM3050S, Leica Biosystems, Deer Park, IL, USA) following the Kawamoto method [34]. Sections for histological analysis were stained with hematoxylin and eosin. Images were acquired with a polarizing microscope (BX41) equipped with a digital camera (DP22, Olympus).

### 2.5. Immunofluorescence

Immunofluorescence staining was performed on the frozen sections. Sections were pretreated with 5% goat serum (Vector Laboratories, Newark, CA, USA) for blocking nonspecific binding and with 0.3% Triton X-100 (Nacalai Tesque, Kyoto, Japan) in phosphate-buffered saline (PBS; FUJIFILM Wako) for 30 min to permeabilize the tissues. Antigen retrieval was performed using Histo VT One (Nacalai Tesque) at 70 °C for 20 min. The sections were then treated for 1 h at room temperature with primary antibodies against CD31, p21, p16, and VEGF, as listed in Table 1. To visualize nuclei by staining with 4′,6-diamidino-2-phenylindole (DAPI), specimens were mounted with DAPI-Fluoromount-G^®^ (Southern Biotechnology, Birmingham, AL, USA). Cell death was analyzed by TUNEL staining using a CF^®^ Dye TUNEL Assay Apoptosis Detection Kit (Cat. #30074; Biotium, Fremont, CA, USA). Fluorescence images were obtained using an all-in-one fluorescence microscope (BZ-X800; Keyence, Osaka, Japan). Semi-quantitative signal analysis was performed using ImageJ software (v2.1.0; National Institutes of Health, Bethesda, MD, USA), focusing on the PDL. The region of interest (ROI) on the pressure side of the PDL was manually delineated based on anatomical landmarks, including the root surface and the alveolar bone surface, and the same criteria were consistently applied across all samples. Positive signals were defined using an intensity threshold above background fluorescence, determined from regions lacking specific staining and applied consistently across all images. Area-based quantification was applied to immunofluorescence signals to avoid variability caused by unclear cellular boundaries and to account for the intra- and extracellular distribution of the target molecules. In addition, to assess the number of cells within the analyzed regions, the number of DAPI-positive nuclei was quantified separately. For overlapping analyses, the extent of signal overlap between two markers was quantified as the percentage of the double-positive area relative to the reference marker–positive area (area/area, %).

### 2.6. Statistical Analysis

All statistical analyses were conducted in Prism software (version 9.5.0; GraphPad Software, Boston, MA, USA). Data are expressed as the mean ± standard deviation (SD). Data normality was assessed using the Shapiro–Wilk test, and homogeneity of variances was evaluated using Brown–Forsythe test. When both assumptions were satisfied, statistical significance was determined using one-way analysis of variance (ANOVA) followed by Tukey’s post hoc test. For datasets in which normality was satisfied but homogeneity of variances was not met, Welch’s ANOVA followed by the Games–Howell multiple comparison test was applied. Datasets that did not satisfy the assumption of normality or were not suitable for Welch’s ANOVA were analyzed using the Kruskal–Wallis test followed by Dunn’s multiple comparison test. A *p*-value less than 0.05 was considered statistically significant.

## 3. Results

### 3.1. Establishment of OTM Model

**Figure 2:**
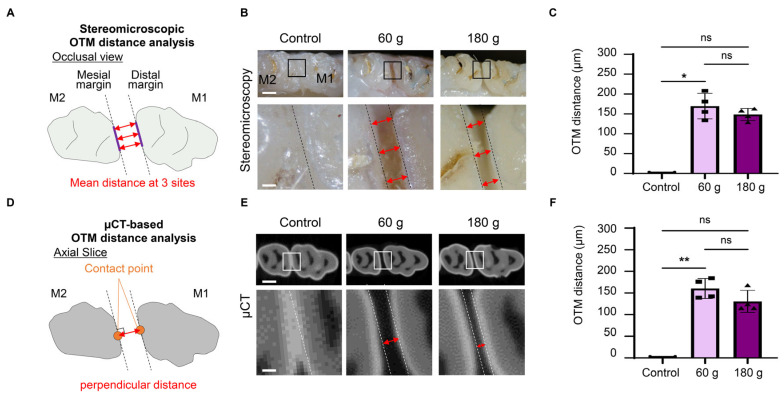
Establishment of the orthodontic tooth movement (OTM) model. (**A**) Schematic illustration of the mean distance measurement using stereomicroscopic images. M1: maxillary first molar, M2: maxillary second molar. (**B**) Representative stereomicroscopic images of OTM in the occlusal view. Red arrows: the distances measured at the three points. (**C**) Quantitative analysis of OTM distance based on stereomicroscopic images. (**D**) Schematic illustration of the perpendicular distance measurement between the contact points of M1 and M2 using micro-computed tomography (µCT) images. (**E**) Representative µCT images of OTM in the axial slice. Double-headed arrows: the perpendicular distance between the contact points of M1 and M2. (**F**) Quantitative analysis of OTM distance based on µCT images. Scale bars: 1 mm (low magnification) and 100 µm (high magnification) in (**B**,**E**). Data are mean ± SD (n = 4). ns: not significant, * *p* < 0.05; ** *p* < 0.01; Kruskal–Wallis with Dunn’s post hoc test.

To analyze the OTM distance in detail, tooth movement was evaluated using two methods: stereomicroscopy and µCT. In the stereomicroscopic images, the average OTM distance was measured between the M1 and the M2 at three points: the buccal and palatal edges and the midpoint (Figure 2A). Stereomicroscopy demonstrated tooth displacement in both the 60 g and 180 g force groups (Figure 2B). There was no statistically significant difference in tooth movement between the 60 g and 180 g groups (Figure 2C). In µCT images, the OTM distance was measured as the perpendicular distance between the contact points of M1 and M2 (Figure 2D). µCT imaging demonstrated no significant difference in the mean OTM differences between the 60 g and 180 g groups (Figure 2E,F), consistent with the stereomicroscopic findings. These findings indicated the successful establishment of the OTM model under two different force magnitudes.

### 3.2. Histological Changes in the PDL During OTM

**Figure 3:**
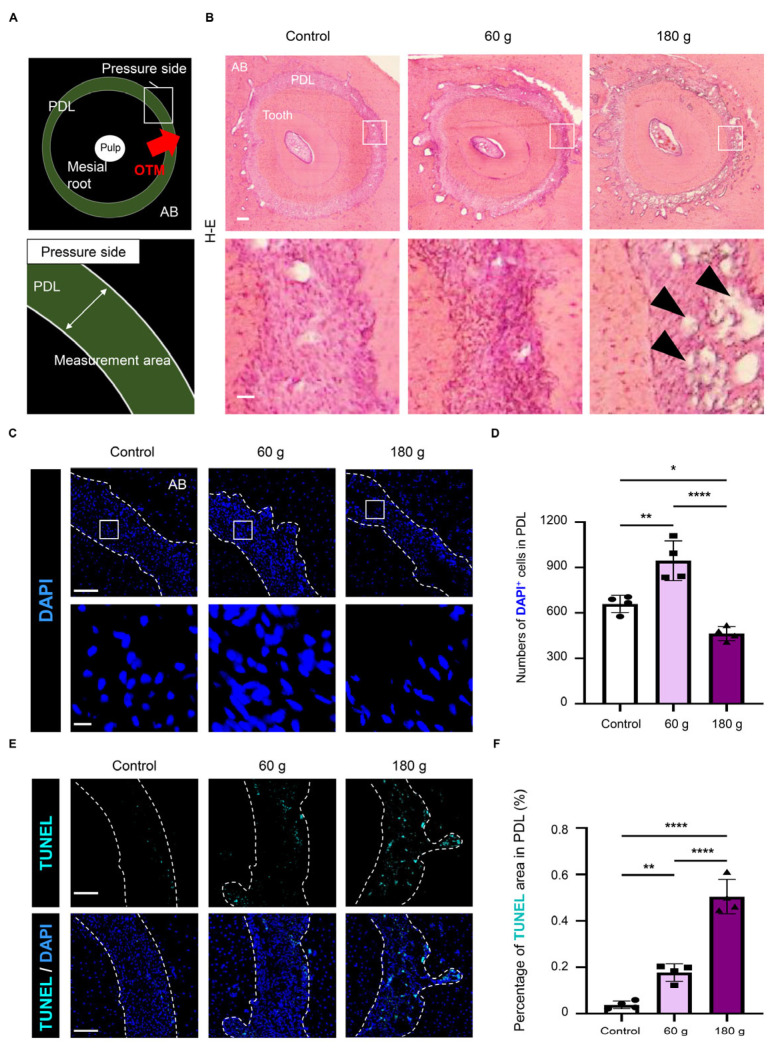
Histological changes in the PDL during OTM. (**A**) Schematic illustration of the measurement area for histological analysis on the pressure side (PS) of the periodontal ligament (PDL) in mesial root. (**B**) Representative hematoxylin-eosin images. Black arrowhead: irregular PDL spaces. Scale bars: 200 µm (low magnification) and 40 µm (high magnification) in (**B**). (**C**) Representative 4′,6-diamidino-2-phenylindole (DAPI) staining images. (**D**) Quantitative analysis of DAPI^+^ cells per total PDL area. (**E**) Representative low-magnification images showing TUNEL and merged TUNEL/DAPI staining. (**F**) Quantitative analysis of TUNEL^+^ area in PDL. Scale bars: 100 µm (low magnification), 10 µm (high magnification) in (**C**,**E**). Data are mean ± SD (n = 4). * *p* < 0.05; ** *p* < 0.01; **** *p* < 0.0001; one-way ANOVA with Tukey’s test. AB: alveolar bone.

To evaluate the histological changes during OTM, the PDL on the PS of the mesial root was defined as the region of interest (ROI), as shown in Figure 3A. Histological examination revealed the presence of irregular PDL spaces (black arrowhead) within the PDL of the 180 g group, whereas such areas were infrequently observed in the 60 g group (Figure 3B). DAPI staining revealed that cellularity in PDL increased in the 60 g group compared with the control group, whereas it decreased in the 180 g group (Figure 3C,D). TUNEL staining demonstrated that cell death was significantly increased in the 180 g group compared with the 60 g group (Figure 3E,F). These findings indicate that the OTM models established with two different forces exhibited distinct histopathological responses depending on the force magnitude. 

### 3.3. Angiogenic Responses During OTM

**Figure 4:**
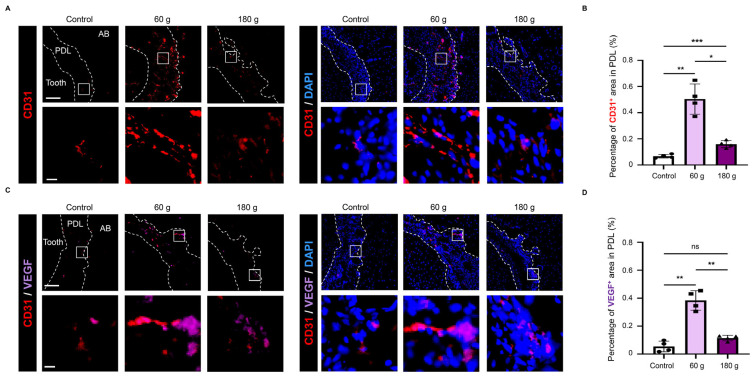
Dynamic changes in angiogenesis induced by OTM. (**A**) Representative CD31 immunofluorescence staining images. CD31 staining without DAPI (**left**) and merged images with DAPI (**right**) are shown. (**B**) Quantitative analysis of CD31^+^ area in PDL. (**C**) Representative CD31/vascular endothelial growth factor (VEGF) double immunofluorescence staining images. CD31/VEGF staining without DAPI (**left**) and merged images with DAPI (**right**) are shown. (**D**) Quantitative analysis of VEGF^+^ area in PDL. Scale bars: 100 µm (low magnification), 10 µm (high magnification). Data are mean ± SD (n = 4). ns: not significant, * *p* < 0.05; ** *p* < 0.01; *** *p* < 0.001; Welch’s ANOVA with Games–Howell post hoc test. PDL: periodontal ligament, AB: alveolar bone.

To evaluate angiogenic responses in the OTM model, immunofluorescence analysis was performed on the PS. CD31 immunostaining demonstrated a significant increase in angiogenic activity in the 60 g group relative to the 180 g group (Figure 4A,B). VEGF immunostaining confirmed elevated VEGF expression in the 60 g group compared with the 180 g group (Figure 4C,D). These results suggest that 60 g of force promoted enhanced angiogenesis and VEGF expression on the PS compared with 180 g.

### 3.4. Senescent Cells During OTM

**Figure 5:**
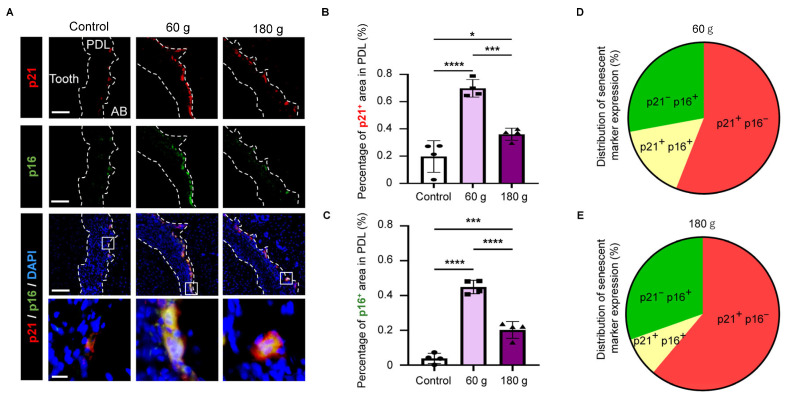
Presence and distribution of senescent cells during OTM. (**A**) Representative p21 and p16 immunofluorescence staining images with DAPI. (**B**) Quantitative analysis of p21^+^ area in PDL. (**C**) Quantitative analysis of p16^+^ area in PDL. (**D**,**E**) Distribution of senescence marker–positive areas in the 60 g and 180 g groups. Scale bars: 100 µm (low magnification), 10 µm (high magnification). Data are mean ± SD (n = 4). * *p* < 0.05; *** *p* < 0.001; **** *p* < 0.0001; one-way ANOVA with Tukey’s test. PDL: periodontal ligament, AB: alveolar bone.

To examine the accumulation and localization of senescent cells under different force magnitudes, immunofluorescence staining for senescence markers was performed on the PS of the OTM model; p21 (representing early-stage senescence) and p16 (representing late-stage senescence) were used. Immunofluorescence staining demonstrated increased p21 and p16 signals in both the 60 g and 180 g groups compared with the control group (Figure 5A). Quantitative area-based analysis revealed that the p21^+^- and p16^+^-positive areas in the PDL were significantly greater in the 60 g group than in the 180 g group (Figure 5B,C). The distribution analysis shows that early senescent cells (p21^+^p16^−^) were predominant, although late senescent cells (p21^+^p16^+^ or p21^−^p16^+^) were also evident (Figure 5D,E).

### 3.5. VEGF Expression of Senescent Cells During OTM

**Figure 6:**
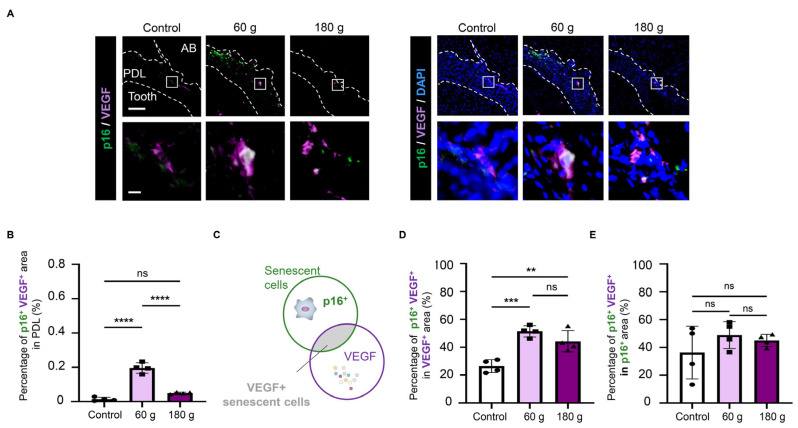
Vascular endothelial growth factor (VEGF) expression of senescent cells during OTM. (**A**) Representative p16 and VEGF double immunofluorescence staining images. p16/VEGF staining without DAPI (**left**) and merged images with DAPI (**right**) are shown. (**B**) Quantitative analysis of p16^+^VEGF^+^ area in PDL. (**C**) Schematic illustration of the quantitative analysis for p16^+^ and VEGF^+^. (**D**) Quantitative analysis of p16^+^VEGF^+^/VEGF^+^ area in PDL. (**E**) Quantitative analysis of p16^+^VEGF^+^/p16^+^ area in PDL. Scale bars: 100 µm (low magnification), 10 µm (high magnification). Data are mean ± SD (n = 4). ns; not significant, ** *p* < 0.01; *** *p* < 0.001; **** *p* < 0.0001; one-way ANOVA with Tukey’s test for (**B**,**D**), and Welch’s ANOVA with Games–Howell post hoc test for (**E**). VEGF: vascular endothelial growth factor, PDL: periodontal ligament, AB: alveolar bone.

To determine whether senescent cells contributed to angiogenic activity through VEGF production, immunofluorescence staining for p16 and VEGF was performed on the PS of the OTM model. Dual immunostaining identified p16^+^VEGF^+^ cells (Figure 6A), with a greater double-positive area observed in the 60 g group than in the 180 g group (Figure 6B). Analysis of the proportion of senescent cells among VEGF-expressing regions (Figure 6C) revealed that more than 40% of VEGF-expressing areas were senescent in both the 60 g and 180 g groups (Figure 6D). In addition, analysis of VEGF expression in senescent regions revealed that over 35% of VEGF-positive areas were senescent in both groups (Figure 6E). Thus, VEGF-expressing senescent cells were detected in the OTM model. 

### 3.6. Senescent ECs Induced During OTM

**Figure 7:**
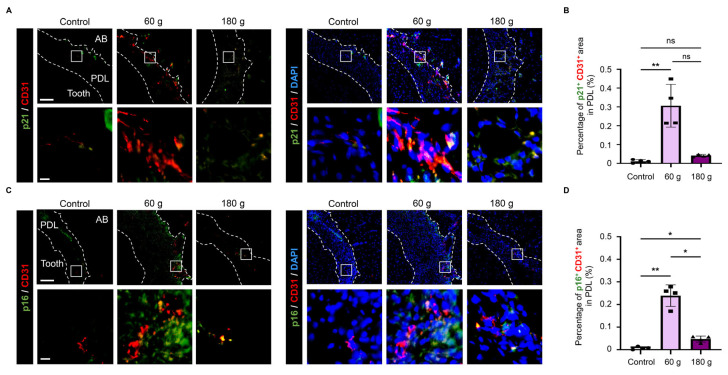
Cellular senescence of vascular endothelial cells (ECs) induced by OTM. (**A**) Representative p21 and CD31 immunofluorescence staining images. p21/CD31 staining without DAPI (**left**) and merged images with DAPI (**right**) are shown. (**B**) Quantitative analysis of p21^+^CD31^+^ area in PDL. (**C**) Representative p16 and CD31 immunofluorescence staining images. p16/CD31 staining without DAPI (**left**) and merged images with DAPI (**right**) are shown. (**D**) Quantitative analysis of p16^+^CD31^+^ area in PDL. Scale bars: 100 µm (low magnification), 10 µm (high magnification). Data are mean ± SD (n = 4). ns: not significant, * *p* < 0.05; ** *p* < 0.01; Kruskal–Wallis with Dunn’s post hoc test. PDL: periodontal ligament, AB: alveolar bone.

To determine whether endothelial cells underwent senescence during OTM, co-immunofluorescence staining was performed using p21 and p16 as senescence markers and CD31 as a vascular endothelial marker. The double-positive cells (p21^+^CD31^+^ and p16^+^CD31^+^) were clearly detected (Figure 7A–D). The abundance and proportions of these senescent ECs were significantly higher in the 60 g group than in the 180 g group (Figure 7B,D). These findings indicate the presence of senescent ECs during OTM.

### 3.7. Overlapping Analysis of Senescent and Endothelial Markers During OTM

**Figure 8:**
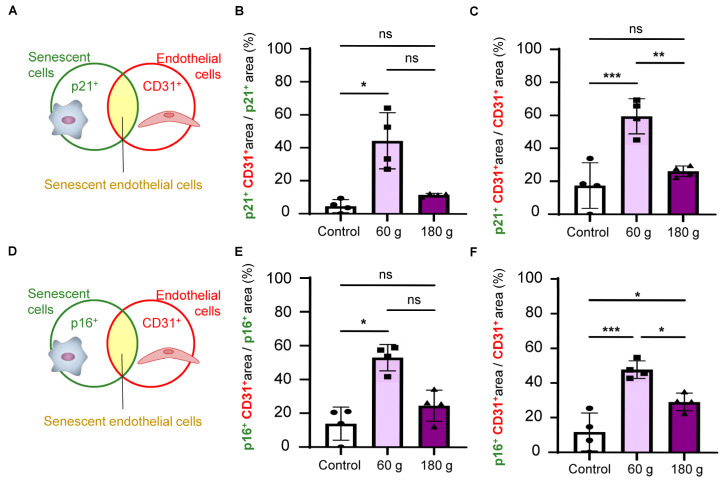
Overlapping analysis of senescent and endothelial markers during OTM. (**A**) Schematic illustration of the overlapping analysis for p21 and CD31 in PDL. (**B**) Quantitative analysis of p21^+^CD31^+^ area/p21^+^ area. (**C**) Quantitative analysis of p21^+^CD31^+^ area/CD31^+^ area. (**D**) Schematic illustration of the overlapping analysis for p16 and CD31 in PDL. (**E**) Quantitative analysis of p16^+^CD31^+^ area/p16^+^ area. (**F**) Quantitative analysis of p16^+^CD31^+^ area/CD31^+^ area. Data are mean ± SD (n = 4). ns: not significant, * *p* < 0.05; ** *p* < 0.01; *** *p* < 0.001; Welch’s ANOVA with Games–Howell post hoc test for (**B**), one-way ANOVA with Tukey’s test for (**C**,**F**), and Kruskal–Wallis with Dunn’s post hoc test for (**E**).

To assess (i) the extent to which OTM-induced senescence was localized to vascular cells, the overlap of endothelial markers within senescent marker–positive areas was analyzed, and (ii) the extent of senescence within endothelial cells under OTM-associated mechanical loading, the overlap of senescent markers within endothelial marker–positive areas was evaluated (Figure 8A–F). In the 60 g group, CD31^+^ overlapped more than 20% of p21^+^ area (Figure 8B), and p21^+^ accounted for over 40% of the CD31^+^ area (Figure 8C). Similarly, CD31^+^ overlapped more than 40% of the p16^+^ area (Figure 8E), and p16^+^ represented more than 40% of the CD31^+^ area (Figure 8F). The extent of overlap was greater in the 60 g group than in the 180 g group (Figure 8B,C,E,F). These results suggest that, in the OTM model, senescent markers are partially localized to endothelial regions and that cellular senescence is exhibited within endothelial cells.

### 3.8. VEGF-Expressing Senescent ECs During OTM

**Figure 9:**
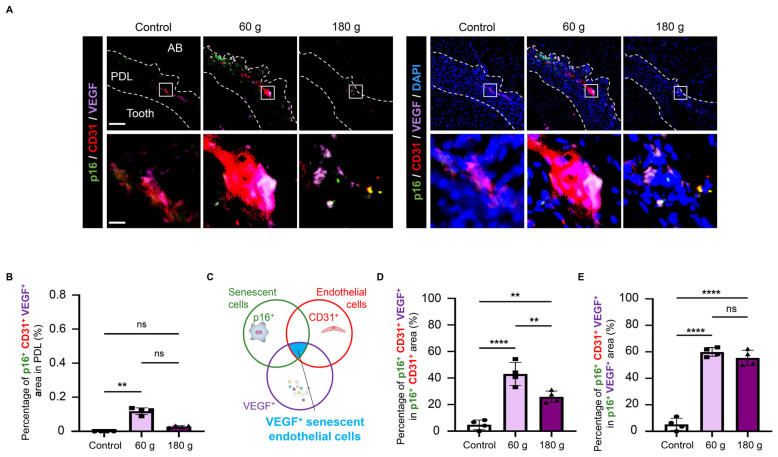
VEGF-expressing senescent ECs. (**A**) Representative p16, CD31, and VEGF triple immunofluorescence staining images. p16/CD31/VEGF staining without DAPI (**left**) and merged images with DAPI (**right**) are shown. (**B**) Quantitative analysis of p16^+^CD31^+^VEGF^+^ area in PDL. (**C**) Schematic illustration of the quantitative analysis for p16, CD31, and VEGF in PDL. (**D**) Quantitative analysis of p16^+^CD31^+^VEGF^+^ area/p16^+^CD31^+^ area. (**E**) Quantitative analysis of p16^+^CD31^+^VEGF^+^ area/p16^+^VEGF^+^ area. Scale bars: 100 µm (low magnification), 10 µm (high magnification). Data are mean ± SD (n = 4). ns: not significant, ** *p* < 0.01; **** *p* < 0.0001; Kruskal–Wallis with Dunn’s post hoc test for (**B**), and one-way ANOVA with Tukey’s test for (**D**,**E**). VEGF: vascular endothelial growth factor, PDL: periodontal ligament, AB: alveolar bone.

To investigate the VEGF expression of these senescent ECs, triple immunofluorescence staining (p16, CD31, and VEGF) was performed, revealing p16^+^CD31^+^VEGF^+^ triple-positive cells (Figure 9A,B). (i) To assess the cellular composition of VEGF+ senescent cells, we analyzed the proportion of CD31^+^ cells within the VEGF^+^ senescent cell population. Furthermore, (ii) to evaluate whether senescent ECs represent a cell population potentially associated with angiogenesis through autocrine mechanisms, we analyzed the proportion of VEGF^+^ cells within the senescent EC population (Figure 9C). Quantitative analysis of VEGF-expressing senescent ECs showed that these cells comprised more than 30% of the total senescent ECs population in the 60 g group, which was higher than that in the 180 g group (Figure 9D). Additionally, ECs accounted for approximately half of the VEGF-expressing senescent cell population in both groups (Figure 9E). These findings demonstrate the presence of VEGF-expressing senescent ECs on the PS during OTM, particularly in the 60 g group.

## 4. Discussion

This study investigated the localization and VEGF expression of senescent cells using a horizontal OTM model. Tooth movement was observed under both 60 g and 180 g conditions. Cellularity and angiogenesis increased under the 60 g condition but decreased under the 180 g condition. Similarly, senescent cells expressing p21 or p16 were more abundant in the 60 g group and fewer in the 180 g group. A subset of senescent cells expressed VEGF, and some of these cells were identified as CD31^+^ endothelial cells.

The magnitude of orthodontic force affects both the amount of tooth movement and the extent of periodontal tissue remodeling [1,5,35]. The study addresses an underexplored concept: the role of senescent cells, particularly senescent ECs, in angiogenesis during OTM. In a coil spring–induced OTM model, Zheng et al. reported that forces between 40 and 80 g effectively promoted tooth movement with minimal side effects, whereas forces exceeding 80 g caused tissue damage, including root resorption, without a corresponding increase in tooth movement distance [3]. In our OTM model using two force magnitudes (60 g and 180 g), histological changes were analyzed during tooth movement (Figure 3). Consistent with previous reports, increasing the orthodontic force from 60 g to 180 g did not lead to a further increase in tooth movement distance (Figure 2). Under the 60 g condition, cellularity (Figure 3) and angiogenic activity (Figure 4) increased markedly, whereas both decreased under the 180 g condition (Figure 3 and Figure 4). In line with previous reports showing that optimal orthodontic forces promote tooth movement and angiogenesis [3,14,16,35], whereas excessive forces suppress both processes [13,14,36]. Consistent with these observations, the 60 g group represented an optimal orthodontic force characterized by increased cellularity and active angiogenesis, whereas the 180 g group was consistent with an excessive force associated with reduced cellularity and suppressed angiogenesis.

Mechanical stress has been reported to induce cellular senescence [17,18,37]. Consistently, mechanical stress generated by vertical orthodontic forces induces cellular senescence in rat models [19]. Similarly, our horizontal rat OTM model demonstrated increased induction of senescent cells under both the 60 g and 180 g conditions compared with controls (Figure 5A–C). The population included both early senescent cells and more irreversible late senescent cells (Figure 5D,E). In this study, the early senescence marker p21 exhibited greater changes between the 60 g and 180 g conditions compared with the late senescence marker p16 (Figure 5B,C). This finding suggests that p21 may respond to mechanical stimulation at an earlier stage, whereas p16, despite showing relatively smaller changes, may reflect a more advanced stage of cellular senescence. Moreover, a greater accumulation of senescent cells was observed under the optimal 60 g force than under the excessive 180 g force. This difference may be explained by the distinct cellular response to mechanical stress: moderate stress allows cells to survive and enter a senescent state, whereas excessive stress causes severe DNA damage and cell death, thereby limiting the accumulation of senescent cells [38]. This interpretation is supported by our observation of reduced cellularity (Figure 3C) and increased TUNEL^+^ areas (Figure 3E) under the excessive 180 g force. Collectively, these findings suggest that mechanical stress partially modulate cell fate—such as senescence or death—in a force-dependent manner during OTM.

Senescent cells play a dual role through the SASP: they promote tissue formation and remodeling during development and wound healing [25,27], whereas they contribute to tissue damage and disease progression under chronic inflammatory conditions [39,40]. Given these dual functions, we examined which aspect of senescent cell activity predominates in the OTM model. The 60 g group exhibited a greater accumulation of senescent cells than the 180 g excessive-force group (Figure 5), paralleling the trend in angiogenesis (Figure 4). We hypothesized that senescent cells may be related to angiogenesis; VEGF expression analysis revealed that a substantial proportion of senescent cells expressed VEGF (Figure 6), including senescent ECs (Figure 9). These findings suggest that senescent cells under optimal mechanical stress are closely associated with angiogenesis during OTM.

Although this is the first study to highlight the association of senescent cells with angiogenesis during OTM, several limitations should be acknowledged. First, the molecular mechanisms by which orthodontic force is sensed and cellular senescence is induced remain unclear. Second, because this study primarily focused on endothelial cells, the potential contributions of other periodontal ligament–resident cell types, such as fibroblasts and osteoblasts, which may undergo senescence and secrete VEGF or other pro-angiogenic factors, remain unexplored. Third, the impact of senescent cells on OTM likely extends beyond angiogenesis. Fourth, this study did not address the functional distinction between senescent and proliferative endothelial cells, which warrants further investigation. Fifth, because a sham-operated control was not included, potential effects of surgical manipulation or orthodontic appliance placement independent of force application could not be fully excluded. Sixth, the absence of inhibitory interventions in this study precludes direct demonstration of a causal role of VEGF-expressing senescent cells in angiogenesis during orthodontic tooth movement, and future studies using senolytic agents or VEGF inhibitors are warranted. Despite these limitations, this study suggests that senescent cells may be related to force-dependent angiogenesis during orthodontic tooth movement, providing insights into the biological role of cellular senescence in periodontal tissue remodeling.

## 5. Conclusions

This study investigated the influence of orthodontic force magnitude on cellular senescence and angiogenesis using a rat horizontal OTM model. Senescent cells were localized in the PDL on the PS. A greater accumulation of senescent cells and enhanced angiogenesis were observed under optimal force conditions (60 g), whereas a reduced accumulation of senescent cells and suppressed angiogenesis were observed under excessive force conditions (180 g). Under optimal force, senescent cells expressing VEGF were more frequently observed. These findings provide new insights into the potential influence of force magnitude on the presence of VEGF^+^ senescent cells during the angiogenic process in OTM.

## Figures and Tables

**Table 1:** Primary antibodies used in this study.

Target	Marker Type	Manufacturer	Catalog No.	Conjugation	Dilution
CD31	Endothelial cell marker	Bioss Antibodies (Shanghai, China)	bs-0195R	BF647	1:100
p21	Senescence marker	Bioss Antibodies (Shanghai, China)	bs-10129R	AF555	1:100
p16	Senescence marker	Bioss Antibodies (Shanghai, China)	bs-23917R	BF488	1:100
VEGF	Growth factor	Bioss Antibodies (Shanghai, China)	bs-4572R	Cy3	1:100

## Data Availability

Data is contained within the article.

## Abbreviations

OTM	orthodontic tooth movement
μCT	micro-computed tomography
VEGF	vascular endothelial growth factor
PDL	periodontal ligament
PS	pressure side
FGF	fibroblast growth factor
TGF-β	transforming growth factor-β
ECs	endothelial cells
SASP	senescence-associated secretory phenotype
MMPs	matrix metalloproteinases
NiTi	nickel–titanium
M1	maxillary first molar
PFA	paraformaldehyde
M2	maxillary second molar
EDTA	ethylenediaminetetraacetic acid
DAPI	4′,6-diamidino-2-phenylindole
SD	standard deviation
ANOVA	analysis of variance
ROI	region of interest
AB	alveolar bone
